# Neurobiological and clinical effect of metacognitive interpersonal therapy vs structured clinical model: study protocol for a randomized controlled trial

**DOI:** 10.1186/s12888-019-2127-2

**Published:** 2019-06-24

**Authors:** Laura Rosa Magni, Antonino Carcione, Clarissa Ferrari, Antonio Semerari, Ilaria Riccardi, Giuseppe Nicolo’, Mariangela Lanfredi, Laura Pedrini, Maria Cotelli, Luisella Bocchio, Michela Pievani, Roberto Gasparotti, Roberta Rossi, CLIMAMITHE Study group, Roberta Rossi, Laura Rosa Magni, Mariangela Lanfredi, Laura Pedrini, Antonino Carcione, Antonio Semerari, Ilaria Riccardi, Giuseppe Nicolo’, Monica Almici, Rossella Beneduce, Genoveffa Borsci, Chiara Caprioli, Matteo Nodari, Antonio Vita, Stefano Barlati, Laura Laffranchini, Luciana Rillosi, Giuseppe Rossi, Luisella Bocchio, Anna Cattaneo, Nadia Cattane, Giovanni Battista Tura, Stefano Bignotti, Maddalena Speziali, Maria Cotelli, Sandra Rosini, Roberto Gasparotti, Claudia Ambrosi, Lorella Mascaro, Daniele Corbo, Michela Pievani, Giulia Quattrini, Elena Bilotta, Livia Colle, Laura Conti, Donatella Fiore, Alessandra Micheloni, Michele Procacci, Valentina Silvestre

**Affiliations:** 1 Unit of Psychiatry, IRCCS Istituto Centro San Giovanni di Dio Fatebenefratelli, Brescia, Italy; 2 Third Centre of Cognitive Psychotherapy, Rome, Italy; 3 Italian School of Cognitive Psychotherapy (SICC), Rome, Italy; 4 Service of Statistics, IRCCS Istituto Centro San Giovanni di Dio Fatebenefratelli, Brescia, Italy; 5 Dipartimento Salute Mentale Roma 5, Rome, Italy; 6 Neuropsychology Unit, IRCCS Istituto Centro San Giovanni di Dio Fatebenefratelli, Brescia, Italy; 7 IRCCS Istituto Centro S. Giovanni di Dio, Fatebenefratelli, Brescia, Italy; 8 Laboratory Alzheimer’s Neuroimaging and Epidemiology, IRCCS Istituto Centro San Giovanni di Dio Fatebenefratelli, Brescia, Italy; 9 Neuroradiology Unit, Department of Medical and Surgical Specialties, Radiological Sciences, and Public Health, University of Brescia, Brescia, Italy

**Keywords:** Borderline personality disorder, Psychotherapy, Neuroimaging, Metacognition, Emotion dysregulation

## Abstract

**Background:**

Borderline Personality Disorder (BPD) is a complex and debilitating disorder, characterized by deficits in metacognition and emotion dysregulation. The “gold standard” treatment for this disorder is psychotherapy with pharmacotherapy as an adjunctive treatment to target state symptoms.

The present randomized clinical trial aims to assess the clinical and neurobiological changes following Metacognitive Interpersonal Therapy (MIT) compared with Structured Clinical Management (SCM) derived from specific recommendations in APA (American Psychiatric Association) guidelines for BPD.

**Methods:**

The study design is a randomized parallel controlled clinical trial and will include 80 BPD outpatients, aged 18–45 enrolled at 2 recruitment centers. Primary outcome will be the clinical change in emotion regulation capacities assessed with the Difficulties in Emotion Regulation Scale (DERS). We will also investigated the effect of psychotherapy on metacognitive abilities and several clinical features such as BPD symptomatology, general psychopathology, depression, personal functioning, and trait dimensions (anger, impulsivity, alexithymia). We will evaluate changes in brain connectivity patterns and during the view of emotional pictures. A multidimensional assessment will be performed at the baseline, at 6, 12, 18 months. We will obtain structural and functional Magnetic Resonance Images (MRIs) in MIT-Treated BPD (*N* = 30) and SCM-treated BPD (*N* = 30) at baseline and after treatment, as well as in a group of 30 healthy and unrelated volunteers that will be scanned once for comparison.

**Discussion:**

The present study could contribute to elucidate the neurobiological mechanisms underlying psychotherapy efficacy. The inclusion of a multidisciplinary study protocol will allow to study BPD considering different features that can affect the treatment response and their reciprocal relationships.

**Trial registration:**

NCT02370316. Registered 02/24/2015.

**Electronic supplementary material:**

The online version of this article (10.1186/s12888-019-2127-2) contains supplementary material, which is available to authorized users.

## Background

Borderline Personality Disorder (BPD) is one of the most common disorders in clinical settings. Psychotherapy is the primary treatment for BPD, with pharmacotherapy as an adjunctive treatment to target state symptoms [1]. In the last two decades, numerous psychotherapeutic approaches have been proposed for this diagnosis and their effectiveness in reducing symptoms and dysfunctions in behaviors seemed to be well supported, even though data on the improvement in social functioning is less optimistic [2–4]. Furthermore, these specialist treatments seem to have similar effects despite distinct theories and interventions. Moreover, despite progress, how psychological therapies produce this improvement is not fully understood [5]. Neurobiological studies could clarify the mechanism of change of psychotherapy for BPD and this could improve our knowledge of the pathophysiology underlying the disease. In particular, several MRI studies explored the neurobiological correlates of the disorder, showed volume reduction in amygdala and hippocampus [6–9], thickness decrease of the prefrontal cortex [10–12], and volume reduction in various regions of the temporal and parietal lobes [13, 14], as compared with healthy subjects. In the field of fMRI studies, the hyperactivity of the amygdala and hypo-activation of frontal areas in response to emotional stimuli in BPD samples [15] seems to be one of the most robust finding. These results were interpreted as the biological substrate of the core symptoms of the disease and, in particular, emotional dysregulation. The key question is whether psychotherapy is able to impact cerebral structures and functional activities and connectivity.

While the majority of the neuroimaging studies of psychotherapeutic treatments have been conducted on Axis I disorders, such as obsessive–compulsive disorder [15–18], mood disorders [19–21], panic disorder [22], social anxiety disorder [23], specific phobia [24, 25], and posttraumatic stress disorder (PTSD) [26], only a few studies explored the impact of psychotherapy in personality disorder and the most are in the context of Dialectical Behavioral Therapy for BPD. Goodman and colleagues showed BPD patients showed an overall decrease in amygdala after 12 months of DBT while the overall amygdala activation of the HC (healthy control) was comparable at baseline and 1 year follow-up [27].

A previous pilot study on 6 BPD patients showed comparable results, in particular a decreasing hemodynamic response to negative stimuli in the right-sided anterior cingulate, temporal and posterior cingulate cortices as well as in the left insula after a 12-week in-patient treatment program [28]. In another interesting study on a 12-weeks DBT program, patients exhibited reduced activity and increased connectivity in neural networks related to salience processing and emotion regulation after treatment [29, 30]. Preliminarily, some effects on brain structure, in terms of increased gray matter volumes in regions that are critically implicated in emotion regulation and higher-order functions, such as mentalizing, have been described [31].

Furthermore, Perez and colleagues [32] showed that after 1 year of Transference-Focused Psychotherapy [4] BPD patients showed relatively increased activation in dorsal prefrontal (dorsal anterior cingulate, dorsolateral prefrontal, and frontopolar cortices) in relation with treatment effect, and relatively decreased activation in ventrolateral prefrontal cortex and hippocampal after the intervention. Noteworthy, an increased left dorsal anterior cingulate cortex activation resulted positevely correlate to clinical improvement in constraint, while left posterior-medial orbitofrontal cortex/ventral striatum activation and negatively with right amygdala/parahippocampal activation seemed to be positively associated to clinical improvement in affective lability correlated. These results are very intriguing and they gave a great contribution to clarify possible mechanisms associated to clinical changes induced by the psychotherapy. No neuroimaging study has yet assessed the effect of other psychotherapy interventions on neurobiological features, e.g. approaches specifically oriented to increase mentalizing [3] or metacognition [33] that are often compromised in BPD and represent one of the core features in BPD patients. Metacognition, as conceptualized by Semerari [33], is the general capacity to think about thinking. Scarce metacognitive abilities have been associated to the difficulty to deal with interpersonal problems and deficit in using problem-solving strategies and choosing adaptive behaviors [34]. Metacognitive Interpersonal Therapy (MIT) is a cognitive behavior-based psychotherapeutic approach aimed to increase metacognitive abilities in order to improve general personality functioning and to promote better interpersonal relationships [35]. More in detail, MIT is designed to support patients in learning to recognize and integrate different mental states and in improving their ability to solve interpersonal problems using mentalistic knowledge of themselves and others.

The aim of the present study is twofold. The primary outcome will be the change in emotion dysregulation, measured by the Difficulties in Emotion Regulation Scale – DERS, [36, 37] after 12 months of MIT treatment in subjects with BPD. The secondary outcomes will be the effect of MIT on neurobiological (changes in cerebral patterns of activation in response to emotional visual stimuli during fMRI scans) and other clinical features. Lastly, we will study the correlation between patients’ metacognitive profiles and structural and functional brain imaging features.

## Method/design

### Trial design

**Fig. 1:**
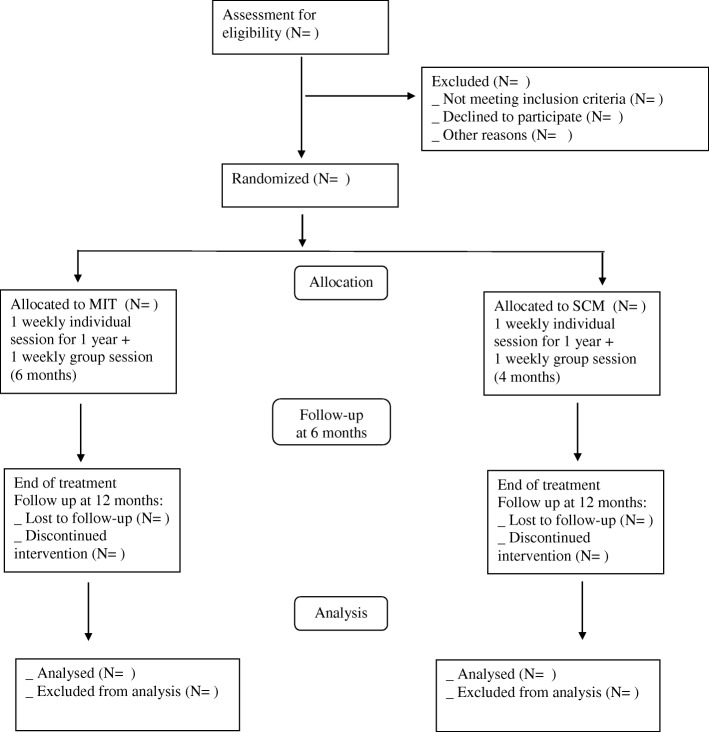
Study Flowchart. MIT: Metacognitive Interpersonal Therapy; SCM Structured Clinical Management

The study design is a randomized parallel controlled clinical trial. For an overview of the proposed flow of participants, see Fig. 1. The present study protocol was written in accordance with the Standard Protocol Items: Recommendations for Interventional Trials (SPIRIT) [38]; copies of the SPIRIT Checklist and figure have been included in Table 1 and Additional file 1.

**Table 1:**
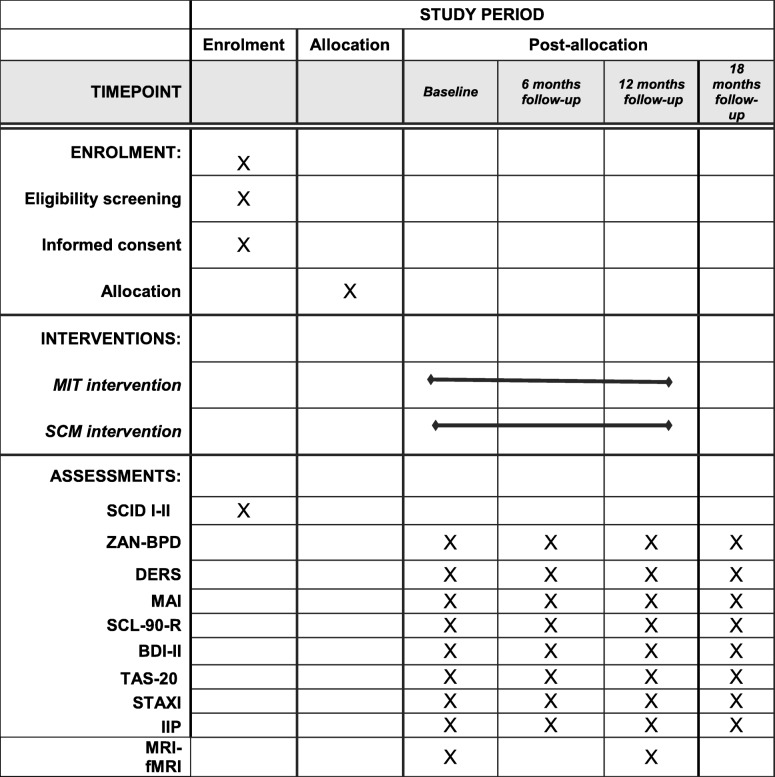
SPIRIT 2013 Figure for CLIMAMITHE study

*MIT* Metacognitive Interpersonal Therapy, *SCM* Structured Clinical Management, *SCID I-II* structured Clinical Interview for DSM disorder, axis I and axis II, *ZAN-BPD* Zanarini Rating Scale for Borderline Personality Disorder, *DERS* Difficulties in Emotion Regulation Scale, *MAI* Metacognitive Assessment Interview, *SCL-90-R* Symptoms Checklist-90- Revised, *BDI-II* Beck Depression Inventory. *TAS-20* Toronto Alexithymia Scale, *STAXI* State and Trait Anger Expression Inventory, *IIP* Inventory of Interpersonal Problems, *MRI-fMRI* Magnetic Resonance Imaging- Functional Magnetic Resonance Imaging

### Participants and study setting

Eighty BPD outpatients will be enrolled at 2 recruitment centres (IRCCS Istituto Centro San Giovanni di Dio, Brescia, Italy; Third Center of Cognitive Psychotherapy, Rome, Italy - Scuola Italiana di Cognitivismo Clinico-SICC, Rome). A group of 30 healthy volunteers will be enrolled as reference group IRCCS Istituto Centro San Giovanni di Dio, Brescia.

### Inclusion and exclusion criteria

The inclusion criteria for patients will be: age 18–45 and a diagnosis of BPD (DSM-IV-TR); informed consent. Patients will be excluded whether a lifetime diagnosis of schizophrenia, schizoaffective disorder, substance abuse or dependence in the 3 months before the enrolment, bipolar disorder, organic mental syndromes, dementia or cognitive impairment, relevant neurological signs will be ascertained. Furthermore, we will exclude pregnant women or lactating or patients receiving concurrent psychotherapy.

HCs will be represented by healthy volunteers without any cognitive impairment or psychiatric/neurologic condition, including alcohol/substance abuse.

### Clinical assessment

Independent expert psychologists will conduct the clinical assessment. Clinical evaluation are scheduled at the baseline, after 6, 12 (end of treatment), and 18 months.

The Structured Clinical Interview for DSM disorder [39, 40] will be used to define the diagnosis and collect data on comorbidities.

### Interventions

Patients will be randomly allocated to one of two interventions: Metacognitive Interpersonal Treatment (MIT) [35] or Structured Clinical Management (SCM) [1] delivered in each recruitment centre.

MIT is a cognitive behavioral-oriented psychotherapy designed to ameliorate metacognitive abilities and to enhance interpersonal relationships [35, 41, 42]. Metacognition pertain to a comprehensive set of cognitive and affective skills aimed to identify mental states, reasoning about them, and attributing them to themselves and others [33, 43]. These skills allow people to understand the reason why other persons react in such way, on the basis of own regularities and constructing personal meaning over their lifespan [44]. MIT is conceptualized to target the general psychopathology of personality. Treatment consists in 50-min one weekly individual session and a metacognitive skill training group (90 min) that cover a period of about 6 months during the year of the treatment. The MIT is manualized and follows a goal hierarchy, as proposed by the authors of the theoretical model [35]. In particular, the main goals of the treatment are: 1) the assessment of symptoms, mental state, metacognitive functions, interpersonal problems, emotion regulation skills; 2) intervention on the aspects that interfere with the therapeutic alliance and/or threaten the patient’s life (in particular, self-harming/suicidal behaviors); 3) intervention on symptoms that cause suffering to the patient; 4) intervention to promote the integration of different mental states MIT sessions will be supervised by the authors of the theoretical model through audio-record and the analyses of the narratives.

SCM is an evidence-based intervention that reflects “best general psychiatric treatment for BPD” and it’s feasible for use by “generalist mental health clinicians” with minimal additional training [45]. It was developed based on “expert consensus” about what general practices work best for treating this condition. SCM is the active comparator used in several study on BPD psychotherapy showing to be effective across an array of clinical outcomes [46]. SCM is tailored on BPD symptomatology and employs a supportive approach with case management and advocacy support. There is an emphasis on psychoeducation, problem-solving, explicit safety planning, medication review and assertive follow-up if appointments are missed. As the comparator, SCM treatment consists in 50-min one weekly individual session and a problem solving group (90 min) that cover a period of about 6 months during the year of the treatment.

Treatment retention was measured by the total number of weeks with at least one session and the number of weeks from the first to the last session attended. Patients were classified as having completed treatment if the time between the first and last sessions was at least 12 months between the 1st and the last session. Dropouts will be defined as those missing four consecutive sessions with no ascertained reason.

Treatment adherence. MIT therapists will be supervised throughout the duration of treatments, and treatment fidelity will be assessed using audiotaped sessions. For each therapist, 2 entire sessions will be recorded and evaluated by 2 senior therapists who developed the MIT, to ensure that the treatment is being done in a rigorous, reproducible, and similar way. As part of the assessment of fidelity, a treatment manual will be developed (as describe above). After each session, each MIT therapist will be required to fulfill a checklist on MIT strategies and techniques in order to monitor the fidelity. For SCM, bimonthly supervision will be mandatory to monitor the case management.

### Therapists’ requirements

Psychotherapies will be delivered by 12 “expert therapists”, with 4-year training in psychotherapy (psychodynamic- or CBT-oriented), a minimum of 2 years of clinical experience and a minimum of 1 year of experience treating BPD patients. More specifically, MIT therapists involved in the project will have received an intensive training in MIT (48 h), 4 years with Cognitive Behavioral are required. For SCM therapists, a 16 h-training on SCM will be delivered, no specific theoretical model will be required but expertise in treating BPD.

In each arm, patients will be randomly assigned to therapists within 2 weeks from the randomization.

### Pharmacological treatment

In order to reduce the possible confounding effect of pharmacotherapy, both on clinical and neuroimaging measures, an adaptation of the APA Guidelines [1] will be adopted to harmonize the prescriptions. Briefly, it includes the following principles: 1) Treatment is symptom specific, directed at particular behavioral dimensions; 2) Affective dysregulation and impulsivity/aggression are risk factors for suicidal behavior, self-injury, and are given high priority in selecting pharmacological agents; 3) Medication targets both acute symptoms (e.g., anger treated with dopamine-blocking agents) and chronic vulnerabilities (e.g., temperamental impulsivity treated with serotonergic agents).

Symptoms to be targeted are divided in three groups: Affective dysregulation symptoms, Impulsive behavioral symptoms and Cognitive-perceptual symptoms and each of this has specific recommendation.

### Primary outcome

The primary outcome measure will be changes in emotion regulation, measured by the Difficulties in Emotion Regulation Scale (DERS) [36, 37]. This scale is a 36-item self-report questionnaire comprising a total score and six dimensions: (1) Non-acceptance of emotional responses (6 items); (2) Difficulties engaging in goal-directed behavior (5 items); (3) Impulse control difficulties (6 items); (4) Lack of emotional awareness (6 items); (5) Limited access to emotion regulation strategies (8 items); (6) Lack of emotional clarity (5 items). The scale showed good psychometric properties, in terms of internal consistency (Cronbach’s alpha between 0.80 and 0.93), construct and predictive validity and adequate test-retest reliability.

### Secondary outcomes

A multidimensional evaluation with standardized tools will assess secondary outcomes in several psychological domains.

Metacognition Assessment Interview (MAI) [33, 47, 48]. Metacognitive functions will be measured with the MAI, a semi-structured clinical interview aimed to evaluate the metacognitive abilities of patients. The interviewers ask for a report of an emotionally meaningful experience or life event occurred in the previous 6 months, in which the subject and another person was involved. After the spontaneous report of the situation, interviewers asked a series of specific questions designed to evaluate the four metacognitive subfunctions (monitoring, integration, differentiation, and decentration).

Symptoms Check-list 90 Revised (SCL-90-R) [49]. General psychopathology will be assessed with the SCL-90-R, a 90 item self-report inventory aimed to measure the presence of psychological symptoms. The SCL-90-R assessed nine symptom dimensions: (1) Somatization; (2) Obsessive–Compulsive; (3) Interpersonal Sensitivity; (4) Depression; (5) Anxiety; (6) Hostility; (7) Phobic Anxiety; (8) Paranoid Ideation; and 9) Psychoticism. For the purpose of our study, we will used the Global Severity Index (GSI), which is the mean value of all of the items, and is considered a measure of global symptomatic distress.

Beck Depression Inventory II (BDI-II) [50]. Depressive symptoms will be evaluated by the BDI-II, a 21-item self-report questionnaire measuring the severity of depressive symptoms in the past 2 weeks with higher scores correlating higher levels of depression.

State-Trait Anger Expression Inventory (STAXI) [51]. State and Trait anger will be assessed with STAXI-2, that is a 57-item inventory which measures the intensity of anger as an emotional state (State Anger) and the disposition to experience angry feelings as a personality trait (Trait Anger). The instrument consists of six scales measuring the intensity of anger and the disposition to experience angry feelings. Items consist of 4-point scales that assess intensity of anger at a particular moment and the frequency of anger experience, expression, and control.

Barratt Impulsiveness Scale (BIS) [52] is a questionnaire aimed to evaluated impulsiveness. The BIS-11 identifies three factors that express three different dimensions of impulsivity: Motor impulsiveness, Impulsivity without planning and Cognitive impulsivity and provides a general index of the construct, as a total score. These scales have shown a good reliability and validity with other measures of the same dimension and the total score of the BIS-11 is an internally consistent measure of impulsiveness.

Interpersonal functioning will be assessed by Inventory of Interpersonal Problems (IIP) [53]. IIP is a 57 items self-report, evaluating different dimensions of interpersonal features (interpersonal sensitivity, interpersonal ambivalence, aggression, need for social approval, and lack of sociability).

Alexithymia will be measured by Toronto Alexithymia Scale (TAS-20) [54], a self-report consisting of 20 items rated on a 5-point Likert scale. TAS-20 provide a total score and three subscales: Difficulties Identifying Feelings; Difficulties Describing Feelings; and Externally Oriented Thinking, which refers to a specific tendency to focus on superficial matters and to avoid emotional thinking (Bagby, et al., 1994).

Childhood traumatic experiences will be evaluated by the Childhood Trauma Questionnaire (CTQ) [55]. The CTQ includes a 28-items that measures 5 types of maltreatment – emotional, physical, and sexual abuse, and emotional and physical neglect.

The attachment experience will be assessed by the Attachment Style Questionnaire (ASQ) [56] that is a 40-item survey that uses a 6-point Likert-type scale. The ASQ yields five factor scores: one is a factor representing secure attachment, the other four represent a particular aspect of insecure attachment.

Data on demographics, suicide attempts, self-injury and aggression episodes, hospitalizations, and pharmacotherapy will be collected.

At the baseline, the neuropsychological test battery included measures used to assess nonverbal reasoning (Raven’s Colored Progressive Matrices), verbal fluency (phonemic and semantic), visuospatial capacity (Rey–Osterrieth Complex Figure Copy), and attention and executive function (Trail Making Test, Wisconsin Card Sorting Test, Stroop Test), memory (Story Recall, Rey–Osterrieth Complex Figure Recall, Digit Span). All of the neuropsychological tests were administered and scored according to standard procedures [57].

Furthermore, all participants completed the Interpersonal Reactivity Index (IRI) [58], the Reading the Mind in the Eyes (RME) [59], the Facially expressed emotion labeling (FEEL) [60] and an experimental task to assess emotional priming (Emotional Priming Paradigm, EPP) [61].

Additionally, we will collect blood samples at baseline and at the different time of observation in order to explore possible peripheral biomarkers of emotional dysregulation and related to the limbic system, neuroplasticity, presence of inflammation and stress response and to observe modifications during treatment.

### Neuroimaging protocol

Structural, functional and diffusion MRI following the ADNI protocol [62] will be acquired in BPD patients twice, at the baseline and after treatment, in order to assess functional and structural brain changes after psychotherapy. Healthy volunteers (*N* = 30) will be scanned once for comparison of baseline data. Image acquisition will be performed on a 3 Tesla scanner with a 64 Channels RF HEAD COIL (Skyra Siemens, Erlangen, Germany) at the Unit of Neuroradiology – Spedali Civili Hospital (Brescia, Italy). In order to assess the cerebral patterns of activation in response to emotional visual stimuli, during fMRI scans (EPI sequence TR/TE 2000/30 ms, voxel size of 2.2 × 2.2 × 3.5 mm), participants will view unpleasant, neutral, and pleasant photographic pictures from the IAPS [63]. In particular, the fMRI study paradigm will be adapted from other studies [27, 64]. Briefly, a total of 96 intermixed unpleasant, neutral, and pleasant photographic images will be presented twice in a random order for a total of 192 trials [27, 64]. Participants will be instructed to watch the picture and then make a three-choice response (unpleasant, neutral, and pleasant) with their dominant hand, basing on the meaning for them personally. Lastly, in order to check that participants have properly understood the task, they will view the same 96 pictures immediately following the scan and will evaluated them using the Self-Assessment Manikin scale (9-point scale) [65].

### Sample size

We consider as primary outcome the DERS score and assume quite homogenous population in terms of DERS scores among the recruitment centers. Previous studies reported that the standard deviation (SD) of DERS was 20.7 in females and 18.8 in males [36]. Thus, we assumed a SD of about 20 points. In addition, the correlation between two evaluations (12 months apart) was expected around 0.7 (indicating that about 50% of the variance of the second measurement should be explained by the first measurement). We computed that the SD of DERS changes will be about 15. Our hypothesis is that MIT will decrease DERS more than SCM and, more precisely, a difference between MIT and SCM mean effect larger than 10 points will be considered clinically relevant. To recognize such difference as statistically significant (at bilateral alpha level of 0.05) with an adequate power (0.80), the total number of patients to be recruited should be 60, raised up to 80 (40 MIT + 40 SCM), considering an attrition rate around 20%.

### Randomization

After baseline assessments, eligible participants will be assigned to treatment arms using randomly generated block randomization scheme within each center. Considering the trade-off between the clinical best practices (e.g. The minimization of waiting list for patients) and rigorous statistical-methodological procedures, block size is fixed equal to 4. With this block size and the two arms (named 0,1), there are 6 different ways -type of blocks Bi- (as result of combination of 4 subjects into 2-element grouping) to allocate the patients: B1 [0011]; B2 [0101]; B3 [0110]; B4 [1010]; B5 [1001]; B6 [1100].

The 80 patients of the project will be randomly assigned to the two arms by a random choice (with replacing) of 20 blocks among the six blocks above defined. For the random choice, the ‘sample’ command of the statistical software R was used (sample (1:6, 20, replace = T)). Random allocation will be done by a statistician. Within 2 weeks, the clinician in charge for clinical evaluation will enrolled the patients and will communicate the experimental arms.

Given the nature of the psychological treatment neither the therapists nor the participants can be blinded for the delivered treatment.

### Data management and storage

Data will be manually entered into a database. Data will be stored at the study site following all the secure procedures: demographical and clinical information will be kept locked in dedicated spaces with limited public access. After obtaining informed consent, each participants will be associated with an alphanumeric unique code. Both the database including demographical and clinical information in an anonymous way and the file containing the name of the participants and their codes will be stored on a secure server and they will be protected by passwords. Only authorized research personnel will be access to the database.

### Statistical analyses

An evaluation of type of missing data will be performed in order to detect any missing not-at-random outcome data. A subsequent data-imputation technique (Bayesian imputation) will be applied to obtain complete outcome data. Descriptive statistics will be carried out with parametric and non-parametric tests accordingly to probability data distribution. The analyses of correlation between metacognitive profiles and specific clinical and morphological brain features (i.e. cortical thickness, regional volumes) will be assessed by linear and/or generalized linear models. For longitudinal analyses we will adopt the generalized linear mixed model or generalized estimating equation (GEE) models based on the covariance structure of the data. Lastly, to identify possible predictors of response, we will adopt logistic regression models where clinical and brain markers will be the covariates and the treatment response will be the dichotomous outcome.

### Neuroimaging analyses

Neuroimaging analyses will aim to 1. assess the structural and functional correlates of BPD, 2. identify biological markers as possible predictors of treatment response, and 3. assess the variations after psychotherapy in cortical and subcortical functional activation in response to a standardized emotional material [63] (Lang et al. 2007) along with structural and functional connectivity. Structural measures will be assessed on MRI using both whole brain cortical thickness analyisis and Region of interest (ROI) analysis of the key subregions involved in emotion regulation (e.g, amygdala). Functional measures will be assessed on task fMRI through a voxel-wise and ROI-Analyses analysis of the fluctuations in the BOLD signal. Structural connectivity analysis will include the assessment of fractional anisotropy, mean, axial and radial diffusivity in the major white matter tracts. The analysis will be carried out both with voxel-wise analysis and ROI approach restricted to the tracts involved in emotion (e.g., limbic tracts).

### Dissemination

Results of the study will be presented at international scientific congresses and published in international scientific journals.

## Discussion

The relevance of the project is twofold. First, the population included in the project represents a clinical priority of mental health system for several reasons (high suicidal risk, high direct and indirect costs, long-term impairment and social dysfunctioning), moreover in Italy there is a paucity of intervention specifically oriented to this clinical group.

Secondly, our project’s contribution will be to test the effectiveness of a psychotherapeutic approach and to identify the clinical and neurobiological factors associated with response to the treatment. The inclusion of a multidisciplinary study protocol will allow to study BPD considering different features that can affect the treatment response and their reciprocal relationships.

The RCT CLIMAMITHE will contribute to deepen this topic, studying in particular the change in emotion dysregulation comparing the two treatments and the relation between these and changes in neurobiological aspects. The rationale to include emotional dysregulation as primary outcome is manifold. From a clinical point of view, emotional dysregulation is one of the core dimensions of BPD and the relationship between emotion regulation capacities and metacognitive abilities has not been yet clearly addressed. In the definition proposed by Gratz and Roemer [36], emotion regulation includes also metacognitive aspects (awareness and understanding of emotions, the ability to control impulsive behaviors and to behave flexibly in accordance with desired goals when experiencing negative emotions). We will investigated the relationship between emotion regulation, metacognitive abilities and other BPD features. From a methodological point of view, DERS has demonstrated good psychometric properties, in terms of internal consistency, test-retest reliability and validity [36, 37]. Lastly, DERS has been previously used to measure clinical and neurobiological change after DBT [27].

The use of the same behavioral task during fMRI used in other study will let the results to be comparable and this could diminish the variabilities of their interpretation. Moreover, the expected number of patients to be included could overcome the limitations of other studies with a small sample.

In addition to ED, another core feature of BPD is the deficit in mentalizing, but no RCT with neuroimaging focused on this variables. By investigating specifically metacognition, this study will permit to contribute to the study on the mechanism of change in BPD treatments and help to provide date to the analisys on “what is expected to change in BPD?” and “which are the specific aspects that mainly contribute to the improvement of the patients’ symptomatology”. In fact also changes in other clinical variables will be investigate and correlate to neuroimaging data.

Moreover, BPD is a heterogeneous diagnosis with numerous comorbidities and different clinical endophenotypes based on subgroups of symptoms (impulsive symptoms, cognitive symptoms, interpersonal symptoms and affective symptoms) [66, 67]. We plan to search for specific pattern in the sample and investigate the relationship with the neurobiological variables.

This is the first RCT on BPD conducted in Italy.

### Strengths and limitations

The main strength of the project is the synergy between clinical and biological expertise. Although psychotherapies are delivered in routine clinical settings, a dedicated research team coordinates and supports the activities of the project. The periodic supervision and the systematic assessment of fidelity delivered by the authors of the MIT is an effort to reduce variability and to maximize the treatment adherence. The multidimensional clinical assessment gives the opportunity to characterize BPD patients.

One possible limitation is represented by the presence (possible but not mandatory) of pharmacotherapy, which could have an impact both on clinical and neurobiological aspects. Although the pharmacotherapy does not represent the first choice in the treatment of BPD patients, it is well documented that pharmacotherapy is very common both in the United States and in Europe, with a percentage of patients prescribed medication ranging between 70% to more than 80% [68–70]. Furthermore, polypharmacy is also a common practice, with more than one-third of participants with BPD in these studies taking at least three drugs. One possible strategy to avoid that the presence of medication represents a potential confounding factor on clinical and neurobiological outcomes could be the enrollment of drug-naïve/free patients. On the one hand, this strategy could be potentially useful, on the other hand it would lead to include a group of patients that might not be representative of the clinical *real world* where BPD patients are commonly treated with pharmacotherapy. We will minimize this source of variability by using a standard methodology to prescribe pharmacotherapy [1]. Furthermore, the large number of subjects will allow us to conduct additional analyses on sub-groups of patients who were given similar treatments. Another critical aspect could be adherence to the psychotherapy, but in the definition of the sample size we considered an attrition rate of 20% to mitigate this problem, coherently with other similar study.

## Trial status

The protocol was registered on ClinicalTrials.gov with the number identifier of NCT02370316. The recruitment started 22/5/2015 and the end of the study is expected on November 2018.

## Additional file

Additional file 1: SPIRIT 2013 Checklist. (DOC 128 kb)

## Abbreviations

DBT: Dialectical Behavioural Therapy
DERS: Difficulties in Emotion Regulation Scale
MAI: Metacognitive Assessment Interview
MIT: Metacognitive Interpersonal Therapy
SCM: Structured Clinical Managment
